# Self-reported hypoglycemia in adult diabetic patients in East Gojjam, Northwest Ethiopia: institution based cross-sectional study

**DOI:** 10.1186/s12902-019-0341-z

**Published:** 2019-01-30

**Authors:** Gashayeneh Genetu Tiruneh, Nurilign Abebe, Getenet Dessie

**Affiliations:** 1 Department of Internal Medicine, School of Medicine, Debre Markos University, P.O. Box 269, Debre Markos, Ethiopia; 2 Department of Public health, College of Health Sciences, Debre Markos University, P.O. Box 269, Debre Markos, Ethiopia; 3 Department of Nursing, College of Medicine and Health Sciences, Bahir Dar University, P.O. Box 79, Bahir Dar, Ethiopia

**Keywords:** Hypoglycemia, Diabetes, Ethiopia

## Abstract

**Background:**

Hypoglycemia presents a barrier to optimum diabetes management and it is related to a negative impact on health-related quality of life, healthcare resource use, and work productivity. Despite the fact that the magnitude of hypoglycemia and factors associated with hypoglycemia in diabetic population were demonstrated in clinical trial settings; there is no adequate evidence concerning to the problem in real-world settings, in particular in the study area. The aim of the study was to assess the magnitude of hypoglycemia and factors associated with hypoglycemia among adult diabetic patients attending chronic follow up clinic at Debre Markos referral hospital, East Gojjam Zone, Northwest Ethiopia, 2017.

**Methods:**

An institution-based cross-sectional study was conducted among 394 sampled diabetic patients who were selected through systematic random sampling technique at Debre Markos referral hospital. Data were collected using structured interviewer-administered questionnaire. The collected data were entered and cleared using epi-data version 3.1 and analyzed by SPSS version 20. We used bivariate and multivariate logistic regression models to identify variables for multivariate analysis and to identify associated factors for hypoglycemia, respectively.

**Result:**

The study revealed that 279(70.8%) of diabetic patients had experienced hypoglycemic event since the diagnosis of diabetes. Patients with type 1 diabetes were more likely to have hypoglycemia as compared with type 2 diabetic patients. The factors found to be significantly associated with hypoglycemia included type 2 diabetes (AOR 0.34, 95%CI: 0.14, 0.82), duration of diabetes from 10 to 14 years (AOR 6.34, 95%CI: 2.12, 18.96) and insulin therapy (AOR 4.93, 95%CI: 2.05, 11.86). Diabetic patients who are government employees (AOR = 0.29, 95%CI: 0.11, 0.78) were less likely to have hypoglycemia when compared to farmers.

**Conclusion:**

The magnitude of hypoglycemia was found to be high and significantly associated with occupation, type of diabetes mellitus, type of medication and duration of diabetes mellitus since diagnosis. Therefore, attention is needed from health-related governmental organizations and health care providers to decrease the burden of hypoglycemia and to address the major contributing factors.

## Background

Diabetes is becoming a pandemic disease resulting in significant morbidity and mortality and an increased need for health care [1–3]. Achieving a target glycemic level is one of the objectives of management of diabetes. The target blood glucose level set for each patient depends on several factors; including the patient’s life expectancy, age, comorbidities, and the impact of hypoglycemia in the patient’s life [4].

Intensification of diabetes control has been given emphasis since the publication of landmark trials such as the Diabetes Control and Complications Trial (DCCT) and the United Kingdom Prospective Diabetes Study (UKPDS) [5, 6]. However, one of the common side effects of intensification of therapy is an increment in the incidence of hypoglycemia [7]. Because of fear of hypoglycemic events, patients may avoid therapy and the glycemic control consequently becomes sub-optimal [4]**.**

There is no single threshold value of plasma glucose concentration which defines iatrogenic hypoglycemia in patients with diabetes; hence it is defined as all episodes of an abnormally low plasma glucose concentration that expose the individual to potential harm [4, 8]. A report from a work group of the American diabetes association and the Endocrine society suggests that patients should be alert at plasma glucose concentration of 70 mg/dl and below [4].

Hypoglycemia has acute short-term symptoms which are related to either counter-regulation, such as tachycardia and sweating, or to neuroglycopenia, such as irritability, confusion, and in severe cases stupor, coma and death [9]. There are also long-term consequences of hypoglycemia such as weight gain, loss of self-confidence, reduced quality of life, decreased working capacity, and increased risk for cardiovascular diseases [4].

Hypoglycemia has considerable impact on the individual’s wellbeing and has a significant cost burden to the healthcare systems and the society [10]. It can cause severe morbidity and even death, depending on its severity or duration [11]. Severe hypoglycemia was associated with a significant increase in adjusted risks of major macrovascular events, major microvascular events, death from a cardiovascular cause, and death from any cause [12].

A literature which reviewed 30 studies (11 real-world data studies and 19 randomized controlled trials) reported that higher rates of hypoglycemia were observed in real-world settings compared with clinical trial settings [8]. Recent prospective studies in European, Asian and African countries on self- reported frequencies of hypoglycemic episodes have shown that hypoglycemia was reported by nearly all of the patients [13, 14]. A review article on hypoglycemia states that the average patient suffers 2 episodes of symptomatic hypoglycemia per week and thousands of such episodes over a lifetime of diabetes [15]. Hypoglycemia occurs about two to three times more frequently in type 1 diabetes than in type 2 diabetes and its incidence increases with the duration of diabetes [8, 16].

In a study of hypoglycemia in diabetic patients in Ethiopia, 61.2% had experienced hypoglycemia since their diagnosis and the factors which showed significant association with hypoglycemia were low educational status, female gender and higher body mass index (BMI) [17].

Awareness of the condition; education of patients, relatives, and health care providers; and selecting an appropriate glucose-lowering medication are some of the strategies to reduce the risk of hypoglycemia [7, 18]**.**

In recent years, there is an increment in the number of diabetic patients with hypoglycemia in Ethiopia. In our clinical practice at Debre Markos referral hospital and Tikur Anbessa specialized hospital, it was observed that there are many diabetic patients with hypoglycemia and some who already have permanent neurologic sequela. However, limited data exist on the frequency of hypoglycemia incidents among diabetic patients in Ethiopia, particularly in the study area. This study was intended to determine the magnitude of hypoglycemia and factors associated with hypoglycemia in adult diabetic patients in the study area.

## Methods

### Study setting

The study was conducted from November 15, 2017 to January 15, 2018, for 8 consecutive weeks at Debre Markos referral hospital; which is located in Debre Markos town, East Gojjam zone, Amhara Regional State, Ethiopia. Debre Markos referral hospital is the only referral hospital in East Gojjam zone. There is a separate follow up clinic for medical patients and two days per week is dedicated to diabetic patients. There are four internists and 10 general practitioners working in the follow-up clinics. Currently, there are about 1100 diabetic patients who are registered and have regular follow up at the clinics [19] .

### Study design and population

Institution-based cross-sectional study design was used to assess hypoglycemia among diabetic patients. Samples were selected from diabetic patients who have follow-up at Debre Markos referral hospital. Patients with gestational diabetes were excluded from the study.

### Sample size and sampling procedure

Sample size calculation for this study was determined using single population proportion formula. The sample size was calculated with the assumption of 61% of patients might have hypoglycemic episode since diagnosis, which was taken from a previous study [17]. After 10% non-response rate consideration, the total sample size for the study was 403.

Most of the diabetic patients are appointed at interval of four to eight weeks. However, for some patients the frequency of their visits can vary depending on their blood glucose level. The total number of diabetic patients who had follow-up from September 15, 2017 to November 15, 2017 was taken to estimate the patient flow. The sampling fraction was calculated by dividing N/n (850/403=2.1), where N = estimated number of patients that will be seen during the study period and n = calculated sample size using single population proportion formula. Then systematic sampling technique was employed to approach the study participants. The first patient was selected by lottery method from the first two patients. Then study participant was selected every two patients until the required sample size is achieved. From the selected diabetic patients, 394 of them gave consent and participated in the study.

### Study variables and measurement

The dependent variable of the study was hypoglycemia. The independent variables were socio-demographic variables, membership to the diabetic association, blood glucose level, type of diabetes mellitus (DM), duration of diabetes, type of treatment, the presence of chronic complications of DM, and presence of other comorbid chronic diseases. Hypoglycemia was defined as an event during which there are typical symptoms of hypoglycemia which are improved after administering carbohydrates, glucagon, or other corrective actions or there is a measured plasma glucose concentration of ≤70 mg/dL (≤3.9 mmol/L) [4]. Severe hypoglycemia was defined as a hypoglycemic event requiring assistance of another person to actively administer carbohydrates, glucagon, or take other corrective actions [4].

### Data collection and quality control methods

Data were collected with structured questionnaire, which was developed through reviewing different literature [4, 17, 20]. The questionnaire had three parts; the first part assessed socio-demographic characteristics of respondents. The second & third part of the questionnaire aimed to assess diabetes-related variables and other diabetes-related complications. The content validity of the questionnaire was examined by seven experts who had more than five-year research and work experience of which two of them from medicine department, three of them from public health department, one from nursing department of Debre Markos university and one of them from internal medicine department of Addis Ababa University. The questionnaire was translated to Amharic and then back to English by language experts, in order to check for consistency and comparability of the finding. A two days training was given to the data collectors and the supervisor, by the principal investigator. Four Bachelor of Science (BSc) professionals from nursing department participated in data collection. For data collection, the pre-tested interviewer-administered structured questionnaire was used to interview the respondents in the follow up clinic. Whenever necessary, the patients’ document was reviewed to ascertain some information like diabetes related complications, blood glucose level, and comorbidities. Diabetes related complications and comorbidities are usually evaluated by the physicians, nurses and optometrists working in the hospital and are documented on the patients chart after bi-annual or annual blood pressure measurement, foot examination, serum creatinine determination and eye examination.

### Data processing and analysis

The collected data were checked for completeness and consistency before the analysis. Data were entered and cleared using epi-data version 3.1 and exported to Statistical Package for Social Sciences (SPSS) version 20 for analysis. Descriptive analysis was done. Logistic regression analysis was fitted to identify the association between dependent and independent variables. The crude and adjusted odds ratios together with their corresponding 95% confidence intervals were computed. A *p*-value < 0.05 and corresponding 95% CI of odds ratio was considered to declare a result as statistically significant. The results were presented using text, tables, and graphs based on the type of the data.

## Result

### Socio-demographic characteristics of participants

A total of 394 diabetes mellitus patients participated in the study. Ninety-one (23.1%) of respondents belonged to age group 45–54 years of age, with median age of 43 (IQR = 29–55) years. Two hundred twenty-eight (57.9%) of the respondents were males and 252(64.0%) were married. Ninety-one (23.1%) of the respondents were farmers. From the total participants, only 26(6.6%) were found to be members of the Ethiopian diabetic association (Table 1).

**Table 1:** Sociodemographic and clinical characteristics of diabetic patients at Debre Markos referral hospital, Northwest Ethiopia 2017(n = 394)

Variable	Characteristics	Number (%)
Sex	Male	228 (57.9)
	Female	166 (42.1)
Age group	18–24	70 (17.8)
	25–34	63 (16)
	35–44	69 (17.5)
	45–54	91 (23.1)
	55–64	59 (15.0)
	≥65	42 (10.7)
Religion	Orthodox Christian	385 (97.7)
	Muslim	9 (2.3)
Marital status	Single	90 (22.8)
	Married	252 (64)
	Divorced	28 (7.1)
	Widowed	24 (6.1)
Residence	Urban	258 (65.5)
	Rural	136 (34.5)
Occupation	Farmer	91 (23.1)
	House wife	64 (16.2)
	Merchant	38 (9.6)
	Government employee	90 (22.8)
	Student	50 (12.7)
	Pensioner	23 (5.8)
	Unemployed	38 (9.6)
Monthly Income (Ethiopian Birr)	≤400	55 (14)
	401–700	55 (14)
	701–999	24 (6.1)
	≥1000	260 (66)
Smoking	Smoker	2 (0.5)
	Ex-smoker	17 (4.3)
	Non-smoker	375 (95.2)
BMI	Underweight	45 (11.4)
	Normal	246 (62.4)
	Over weight	75 (19)
	Obese	28 (7.1)
Member of Ethiopian Diabetic association	Yes	26 (6.6)
	No	368 (93.4)

### Diabetes-related characteristics and chronic complications in participants

Two hundred eleven (53.6%) of the respondents had type 1 diabetes mellitus, and the mean duration of diabetes was 5.5 years (SD ± 5.2 years). Two hundred fifty-three (64.2%) of the participants were taking insulin only and 108(27.4%) were taking oral hypoglycemic agents (OHA) only; 33(8.4%) were taking both OHA and insulin. From type 2 diabetic patients 108(59.02%) were treated with OHA, 54(29.51%) were treated with insulin only and 21(11.46%) were treated with both insulin and OHA. Twenty-two (5.6%) of the respondents had a fasting blood glucose level of less than 70 mg/dl at the day of their visit during data collection. (Table 2).

**Table 2:** Diabetes related characteristics in diabetic patients at Debre Markos referral hospital, Northwest Ethiopia 2017(n = 394)

Variables	Characteristics	Number (%)
Type of Diabetes	Type 1	211 (53.6)
	Type 2	183 (46.4)
Duration of diabetes	< 2 years	89 (22.6)
	2–4 years	137 (34.8)
	5–9 years	98 (24.9)
	10–14 years	46 (11.7)
	≥15 years	24 (6.1)
Type of medication for both types of diabetes	Glibenclamide	5 (1.3)
	Metformin	48 (12.2)
	Glibencamide and Metformin	55 (14)
	NPH insulin only	245 (62.2)
	NPH and Regular insulin	8 (2)
	NPH insulin and Metformin	33 (8.4)
Type of medication for both types of diabetes in summary	OHA	108 (27.4)
	Insulin	253 (64.2)
	OHA and Insulin	33 (8.4)
Type of medication for type 2 diabetes	OHA	108 (59.0%)
	Insulin	54 (29.5%)
	OHA and Insulin	21 (11.5%)
FBS level during visit (mg/dl)	< 70	22 (5.6)
	70–130	173 (43.9)
	131–180	98 (24.9)
	181–250	61 (15.5)
	> 250	40 (10.2)

*mg/dl milligrams per deciliter

Hypertension was documented in 96(24.4%) of participants. Retinopathy was found in 65 (16.5%) of the study participants while other complications and comorbidities including neuropathy, cardiac disease, nephropathy, foot ulcer and sexual dysfunction were documented in 58(14.7%), 10(2.5%), 11(2.8%), 7(1.8%) and 6(1.5%) of the patients’ card, respectively. We used chi-square test or fisher’s exact test to assess statistical significance (Table 3).

**Table 3:** Diabetes related complications in diabetic patients at Debre Markos referral hospital, Northwest Ethiopia 2017(*n* = 394)

Diabetes related complications/comorbidities		Type 1	Type 2	Total	P value
		N (%)	N (%)	N (%)	
Retinopathy	Yes	26 (12.3)	39 (21.3)	65 (16.5)	0.023
	No	185 (87.7)	144 (78.7)	329 (83.5)	
Peripheral neuropathy	Yes	20 (9.5)	38 (20.8)	58 (14.7)	0.003
	No	191 (90.5)	145 (79.2)	336 (85.3)	
Heart disease	Yes	4 (1.9)	6 (3.3)	10 (2.5)	0.524
	No	207 (98.1)	177 (96.7)	384 (94.5)	
Hypertension	Yes	26 (12.3)	70 (38.3)	96 (24.4)	0.001
	No	185 (87.7)	113 (61.7)	298 (75.6)	
Nephropathy	Yes	3 (1.4)	8 (4.4)	11 (2.8)	0.143
	No	208 (98.6)	175 (95.6)	383 (97.2)	
Foot ulcer	Yes	0 (0.0)	7 (3.8)	7 (1.8)	0.004
	No	211 (100.0)	176 (96.2)	387 (98.2)	
Sexual dysfunction	Yes	3 (1.4)	3 (1.6)	6 (1.5)	0.588
	No	208 (98.6)	180 (98.4)	388 (98.5)	

*N Number

### Frequency of hypoglycemia

**Fig. 1:**
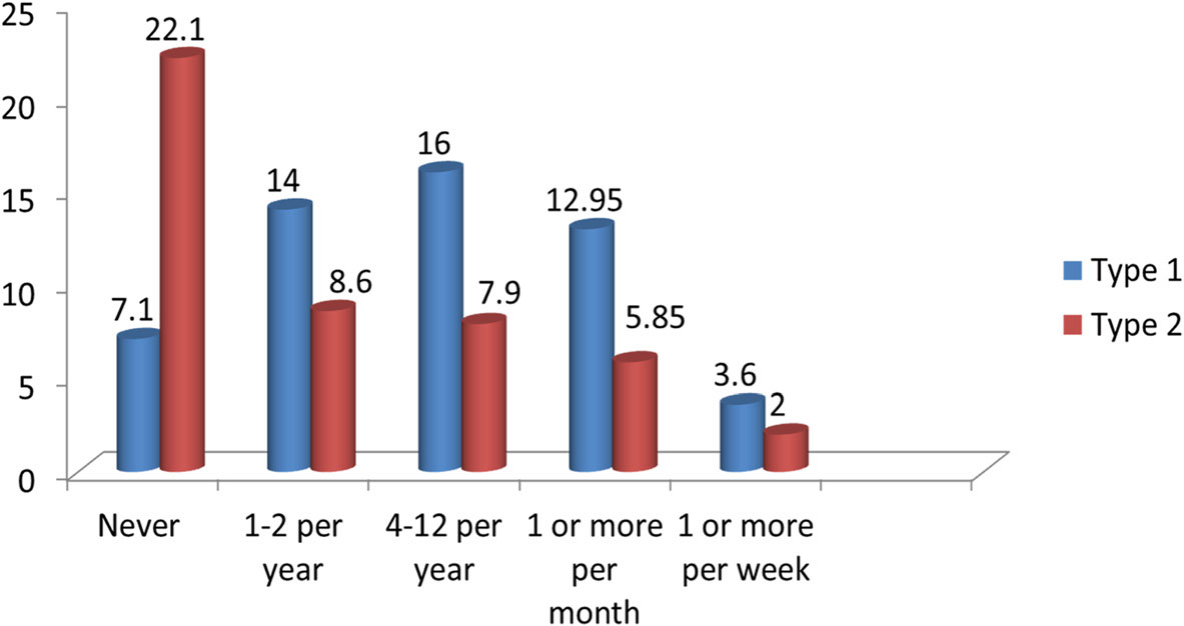
Proportion of diabetic patients with hypoglycemic episodes ever since diagnosis at Debre Markos referral hospital, Northwest Ethiopia 2017(*n* = 394)

Out of the total participants, 279(70.8, 95% CI: 66.8–75.6%) of them had history of hypoglycemia since they were diagnosed with diabetes mellitus. Out of the 211 type 1 diabetic patients, 183(86.7%) of them had history of hypoglycemia; 96(52.5%) of the 183 type 2 diabetic patients had history of hypoglycemia since they were told to have diabetes. The frequency of hypoglycemic episodes were described as never, one to two per year, 4 to 12 per year, 1 or more per month and one or more per week and there was statistically significant difference between type 1 and type 2 diabetes at *p* value of less than 0.001.From the total participants, 7.1% of respondents with type 1 diabetes and 22.1% of respondents with type 2 diabetes have never experienced a hypoglycemic episode (Fig. 1).

A total of 315 episodes of hypoglycemia had occurred since one month prior to the study, out of these 213 episodes occurred in type 1 diabetes patients and 105 episodes occurred in type 2 diabetes patients. This difference in the number of hypoglycemic episodes is statistically significant at *p* value of < 0.05.

Among the total participants, 105(26.6%) had history of severe hypoglycemia out of which 89 (22.6% of the total participants) reported that they had one or more episode of severe hypoglycemia in the preceding year. Only 24 individuals (6.1%) reported that they measure their blood glucose level before exercise or heavy work. Three hundred sixty-nine (93.7%) of the participants have mentioned appropriate corrective actions for hypoglycemia (take sugar, take food or go to a nearby health facility). We used chi-square test or fisher’s exact test to assess statistical significance (Table 4).

**Table 4:** Frequency of hypoglycemic events among diabetic patients at Debre Markos referral hospital, Northwest Ethiopia 2017(*n* = 394)

Variables		Type 1	Type 2	Total	*P* value
		N (%)	N (%)	N (%)	
History of hypoglycemic episode ever since diagnosis of diabetes	Yes	183 (86.7)	96 (52.5)	279 (70.8)	0.001
	No	28 (13.3)	87 (47.5)	115 (29.2)	
History of severe hypoglycemic episode ever since diagnosis	Yes	68 (32.2)	37 (20.2)	105 (26.6)	0.005
	No	143 (67.8)	146 (79.8)	289 (73.4)	
Frequency of hypoglycemia in the last one month	Once	38 (18.01)	22 (12.02)	60 (15.23)	
	Twice	33 (15.64)	15 (8.20)	48 (12.18)	
	3 times	14 (6.64)	7 (3.83)	21 (5.33)	
	4 times	6 (2.84)	2 (1.09)	8 (2.03)	
	5 or more times	7 (3.32)	4 (2.18)	11 (2.79)	
	Total episodes	213	102	315	0.001
Frequency of hypoglycemia in the last one month during sleep	Once	16 (7.58)	4 (2.18)	20 (5.08)	
	Twice	4 (1.90)	3 (1.64)	7 (1.78)	
	3 times	1 (0.47)	2 (1.09)	3 (0.76)	
	4 times	2 (0.95)		2 (0.51)	
	Total episodes	35	16	51	0.024
Frequency of severe hypoglycemia in the past one year	Once	25 (11.85)	12 (6.56)	37 (9.39)	
	Twice	16 (7.58)	7 (3.83)	23 (5.84)	
	3 times	7 (3.32)	7 (3.83)	14 (3.55)	
	4 times	1 (0.47)	2 (1.09)	3 (0.76)	
	5 or more times	9 (4.27)	3 (1.64)	12 (3.05)	
	Total episodes	152	73	225	0.76
How often do you discuss hypoglycemia with physician	Never	35 (16.58)	55 (30.05)	90 (22.84)	0.001
	Sometimes	65 (30.81)	60 (32.77)	125 (31.73)	
	Always	111 (52.61)	68 (37.16)	179 (45.43)	
Corrective actions if/when hypoglycemic	None	3 (1.42)	16 (8.74)	19 (4.82)	0.001
	Take sugar	170 (80.57)	134 (73.22)	304 (77.16)	
	Take insulin	1 (0.47)		1 (0.25)	
	Go to nearby health facility	5 (2.37)	12 (6.56)	17 (4.31)	
	Eat food	32 (15.17)	21 (11.46)	53 (13.45)	
Do you measure your blood glucose level before exercise or heavy work	Yes	12 (5.7)	12 (6.6)	24 (6.1)	0.88
	No	199 (94.3)	171 (93.4)	370 (93.9)	

*N Number

### Factors associated with hypoglycemia

As can be noted from the result of the bivariate logistic regression analysis; age, marital status, residence, occupation, educational status, BMI, type of diabetes, discussion of hypoglycemia with physician, retinopathy, neuropathy, cardiac disease, hypertension, duration of the disease and type of medication did show association with hypoglycemia at *p* value less than 0.25 level of significance.

Consequently, the multivariable logistic regression analysis was used by taking all the 14 factors into account simultaneously and only four of the most contributing factors remained to be significantly and independently associated with hypoglycemia and have an overall significant effect on the outcome variable at 5% level of significance. Occupation, type of diabetes, duration of diabetes and type of medication were found to have significant association with hypoglycemia.

Diabetic patients who are government employees’ were 71% less likely to have hypoglycemia as compared with farmer diabetic patients with (AOR = 0.29, 95%CI: 0.11, 0.78). And diabetic patients who are merchants were 71% less likely to have hypoglycemia as compared with farmer diabetic patients with (AOR = 0.29, 95%CI: 0.09, 0.96).

Concerning with the type of diabetes, patients with type 2 diabetes were 66% less likely to have hypoglycemia as compared with those patients with type 1 diabetes with (AOR = 0.34, 95% CI: 0.14, 0.82).

Diabetic patients with duration of diabetes since diagnosis of 2–4 years (AOR = 3.22, 95% CI:1.58,6.58), 5–9 years (AOR = 3.88,95% CI:1.75,8.61), and 10–14 years (AOR = 6.34,95% CI:2.12,18.96) were more likely to have hypoglycemic episode when compared to those with < 2 years duration of diabetes.

Furthermore, type of medication has shown statistically significant association with the outcome variable. Patients who were taking insulin were 4.93 times more likely to have history of hypoglycemia as compared with those who were taking OHA only with (AOR = 4.93, 95% CI: 2.05, 11.86) (Table 5).

**Table 5:** Factors associated with hypoglycemia in diabetic patients at Debre Markos referral hospital, Northwest Ethiopia, 2017(*n* = 394)

Variables		Hypoglycemia		Crude OR	Adjusted OR	*P* value
		Yes(N)	No(N)			
Occupation	Farmer	81	10	1	1	
	House wife	48	16	0.37 (0.16,0.88)	0.53 (0.19,1.67)	0.300
	Merchant	22	16	0.17 (0.07,0.43)	0.29 (0.09,0.96)	0.042
	Government employee	49	41	0.15 (0.07,0.32)	0.29 (0.11,0.78)	0.015
	Student	35	15	0.29 (0.12,0.70)	0.11 (0.04,0.33)	0.001
	Pensioner	14	9	0.19 (0.07,0.56)	0.57 (0.14,2.35)	0.439
	Unemployed	30	8	0.46 (0.17,1.28)	0.44 (0.12,1.56)	0.201
Type of Diabetes	Type 1	183	28	1	1	
	Type 2	96	87	0.17 (0.10,0.28)	0.34 (0.14,0.82)	0.015
Retinopathy	Yes	51	14	1	1	
	No	228	101	0.62 (0.33,1.17)	0.47 (0.21,1.05)	0.066
Hypertension	Yes	54	42	1	1	
	No	225	73	2.40 (1.48,3.88)	1.89 (0.96,3.72)	0.066
Duration of Diabetes	< 2 years	47	42	1	1	
	2–4 years	100	37	2.42 (1.38,4.24)	3.22 (1.58,6.58)	0.001
	5–9 years	73	25	2.61 (1.41,4.83)	3.88 (1.75,8.61)	0.001
	10–14 years	40	6	5.96 (2.30,15.46)	6.34 (2.12,18.96)	0.001
	≥15 years	19	5	3.40 (1.17,9.89)	2.53 (0.69,9.28)	0.161
Types of medication	OHA	38	70	1	1	
	Insulin	215	38	10.42 (6.17,17.61)	4.93 (2.05,11.86)	0.001
	OHA & insulin	26	7	6.84 (2.72,17.23)	5.35 (1.88,15.28)	0.002
Discussion of hypoglycemia with a physician	Never	56	34	1	1	
	Sometimes	82	43	1.16 (0.66,2.03)	0.76 (0.38,1.57)	0.481
	Always	141	38	2.25 (1.29,3.93)	1.65 (0.79,3.46)	0.182

*N Number

## Discussion

This cross sectional study attempted to estimate the prevalence of hypoglycemia and factors associated with hypoglycemia among adult patients with diabetes mellitus in the study area. The study gives a clue regarding the burden of the problem and the contributing factors. The study showed that hypoglycemia is common in adults with both type 1 and type 2 diabetes. It was found that 70.8% of diabetic patients had one or more hypoglycemic episode in the past and severe hypoglycemic episode was also reported by 26.6% of respondents.

The frequency of hypoglycemia (all episodes and severe episodes) appeared to be higher than has been reported previously [17, 21], suggesting that this may represent a greater clinical problem than has been appreciated. The higher magnitude of hypoglycemia in this study might be due to fact that most of the diabetic patients had type 1diabetes and were on insulin. However, the finding of this study was lower than found in recent large prospective studies on self-reported incidence of hypoglycemia in diabetic patients; in which nearly all patients reported hypoglycemia during the prospective period [13, 14]. The higher frequency of hypoglycemia in prospective studies can be explained by recall bias in self-reporting hypoglycemia in retrospect. The implication of this finding is that there is a high magnitude of hypoglycemia among patients with diabetes which requires active follow up and implementation of prevention strategies.

The study also showed that occupation has a significant association with hypoglycemic episodes. Merchants, government employees, and students are less likely to have hypoglycemia when compared to farmers. This might be due to several reasons. The first reason might be related to the daily activities of the patients. Farming is a much more complex activity which needs high energy expenditure and it may lead to hypoglycemia. The second reason might be related to their living status. In Ethiopia, most farmers are living in the rural area where there are poor infrastructure and transportation. Therefore, follow up clinic may not be easily accessible for such patients. Due to this, patients are likely have poor follow up and poor adherence to diet modification. Hence, health professionals should work with farmers with diabetes to minimize the frequency and the consequences of hypoglycemia. Alternative care facilities (primary health care) with periodic suvervision by senior physicians might be useful for farmers who live in areas with poor infrastructure. Diabetes education and experience sharing through Ethiopian diabetes association among diabetic patients can also help to increase patients’ knowledge and skill on prevention of hypoglycemia.

In this study the proportion of type 1 diabetes is higher than seen in other areas; and this might be explained by 1) the study was conducted in a semi-urban area where most of the patients were poor farmers having diabetes mellitus since childhood 2) the possibility of acquiring viral infections during childhood and 3) the fact that obesity is not common in the study area. This study showed that diabetic patients with type 1 diabetes were more likely to have hypoglycemia than patients with type 2 diabetes. This might be due to the fact that all type 1 diabetes patients are put on insulin therapy which has a higher risk of iatrogenic hypoglycemia [4]. In the study from Austria, self-reported hypoglycemia rate in type 1 respondents was four times greater than the rates reported by type 2 respondents [22]. Other studies have also shown higher frequency of hypoglycemia in type 1 diabetes when compared to type 2 diabetic patients despite being on similar type of treatment [13, 14, 23].

Furthermore, insulin therapy was associated with hypoglycemia. This finding is in accordance with the finding of previous studies which were conducted in Austria, other European countries and African countries [13, 20, 22]. Insulin therapy is one of the most common causes of iatrogenic hypoglycemia [7, 15]. Therefore, patients on insulin therapy need to have closer follow up of their glycemic control.

This study suggests that diabetic patients with increasing duration of diabetes were more likely to have hypoglycemia. A report of a workgroup of the American diabetes association and the Endocrine society states that the incidence of iatrogenic hypoglycemia increases with the duration of diabetes [4]. Several other studies have also shown that longer duration of diabetes increases the risk of hypoglycemia [7, 8, 11]. Therefore, health care workers should give due attention to patients with longer duration after diagnosis of diabetes.

### Limitations of the study

There are limitations to this study. First, there may be recall bias of hypoglycemic episodes since it is a self-reported data. On the other hand, some of the patients didn’t confirm all of the episodes of hypoglycemia with capillary glucometer; so they could also be reporting symptoms without having a blood glucose level of below 70 mg/dl. It is also worth mentioning the limitation of accuracy and availability of the diagnostic methods routinely employed to make the diagnosis of chronic complications of diabetes. Therefore, the findings of this study would be best if it is interpreted in the context of such inevitable limitations of the study.

## Conclusion

The magnitude of hypoglycemia was found to be high and significantly associated with occupation, type of diabetes mellitus, duration of diabetes mellitus since diagnosis, and type of medication. Therefore, attention is needed from health-related governmental organizations and health care providers to decrease the magnitude of hypoglycemia and to address the major contributing factors.

## Abbreviations

ADA: American diabetes association
BMI: Body mass index
DCCT: Diabetes control and complications trial
DM: Diabetes Mellitus
NCDs: Non communicable diseases
OHA: Oral hypoglycemic agent
